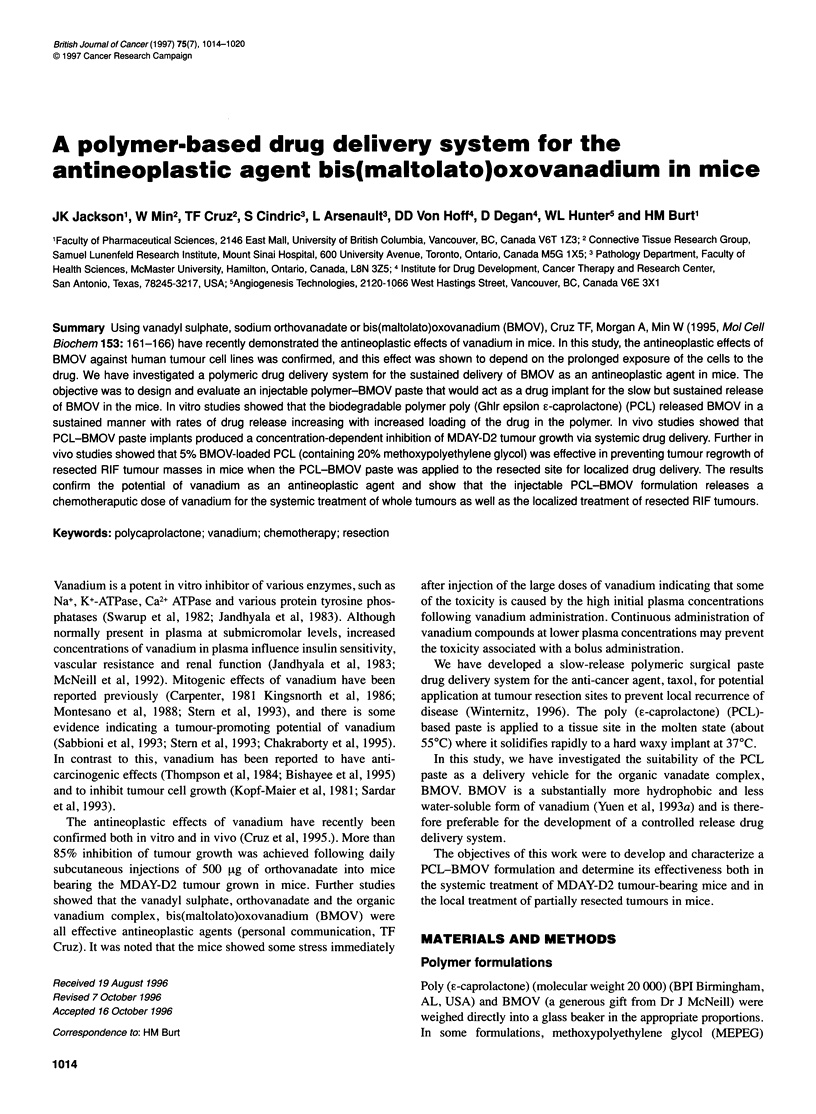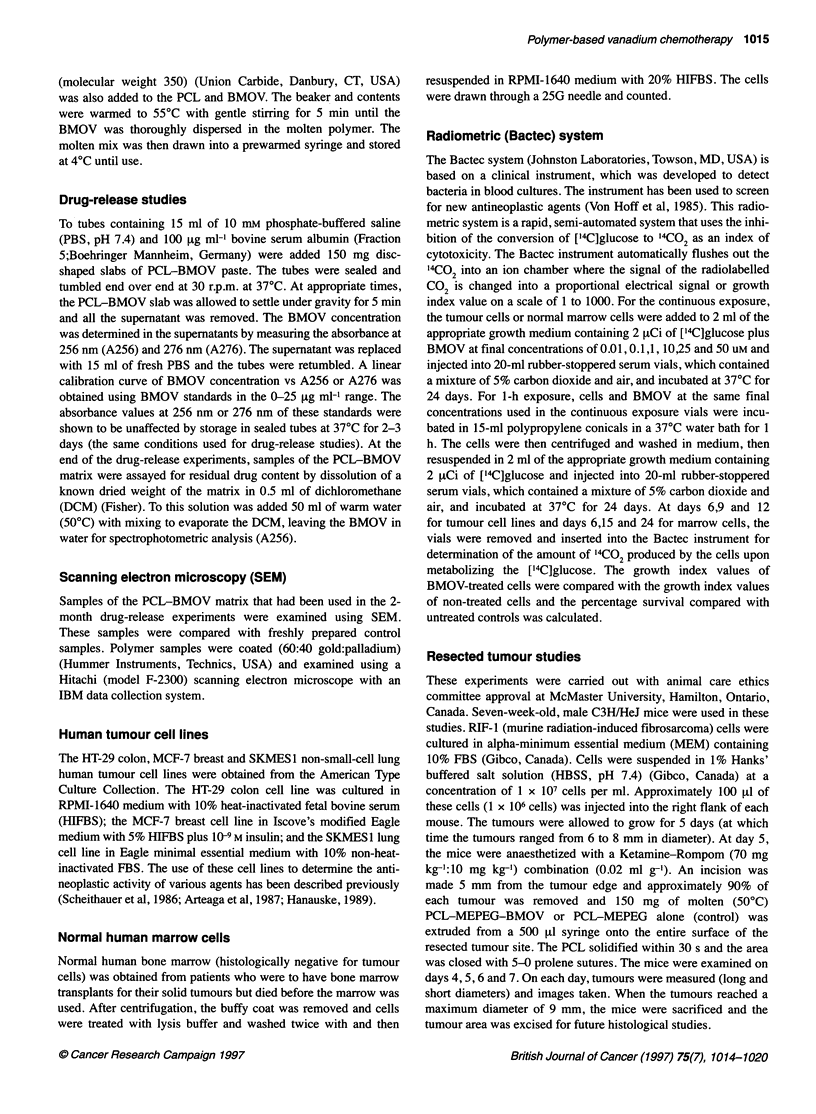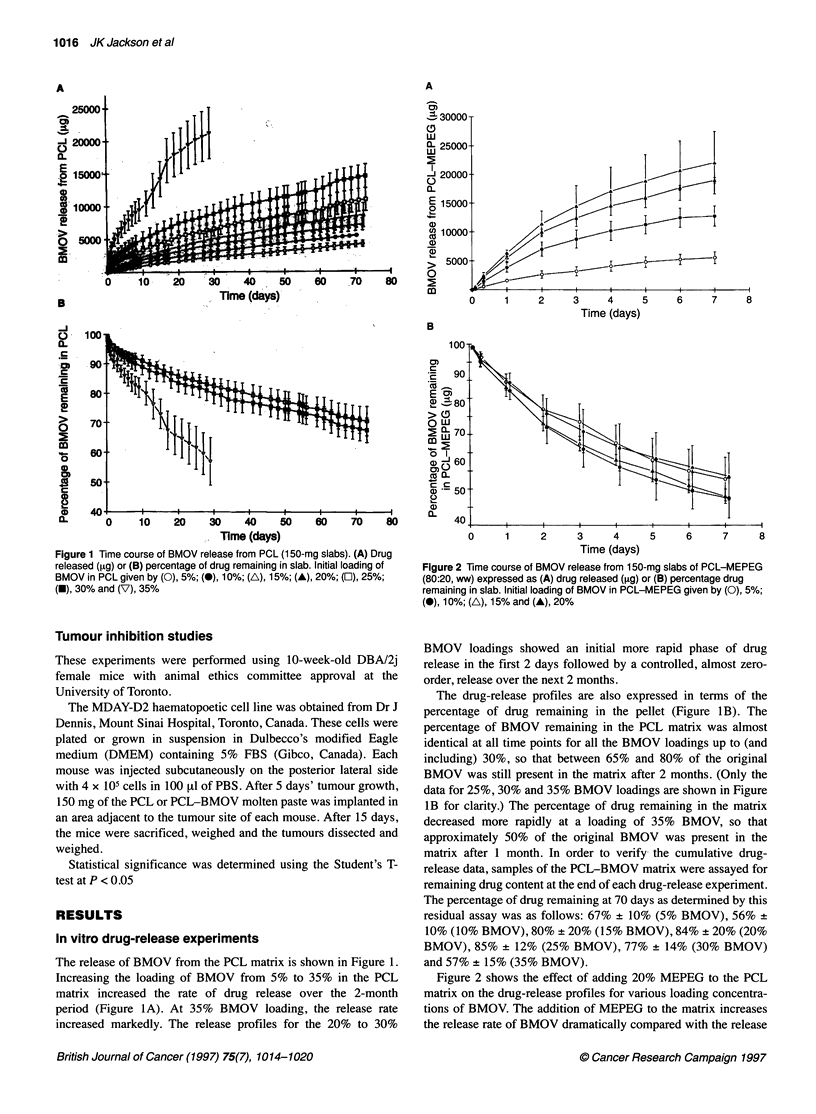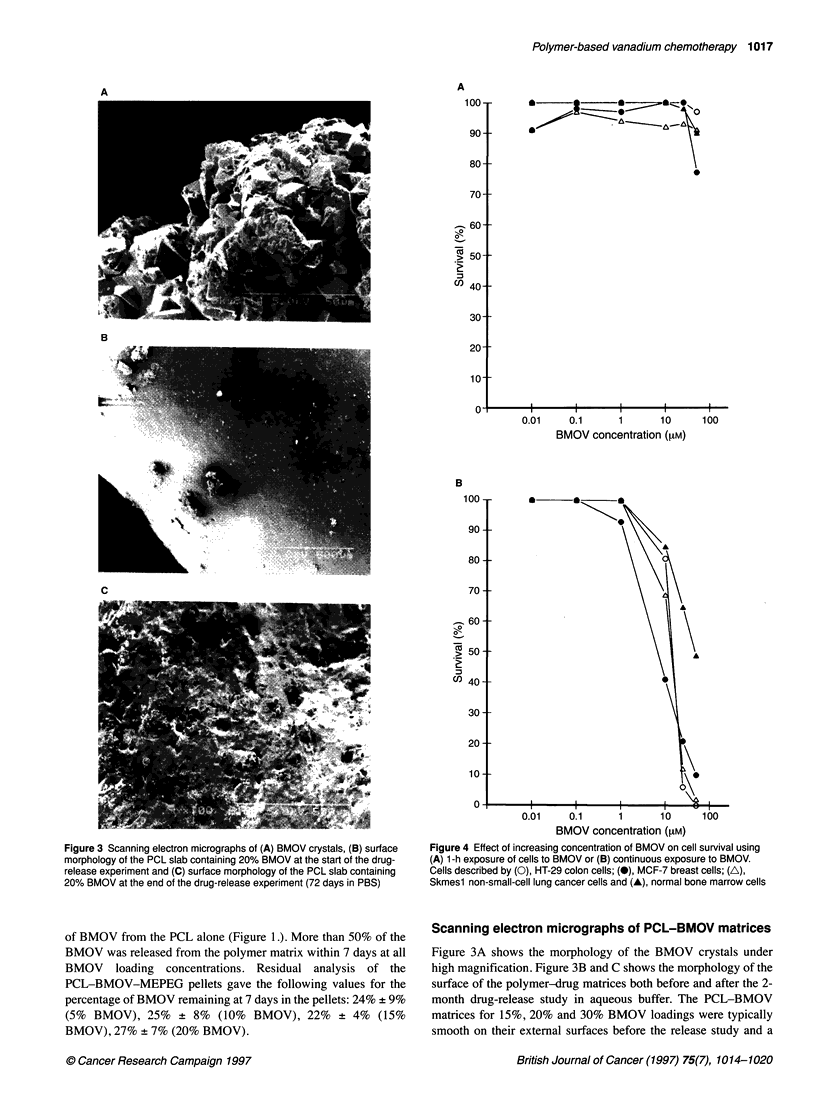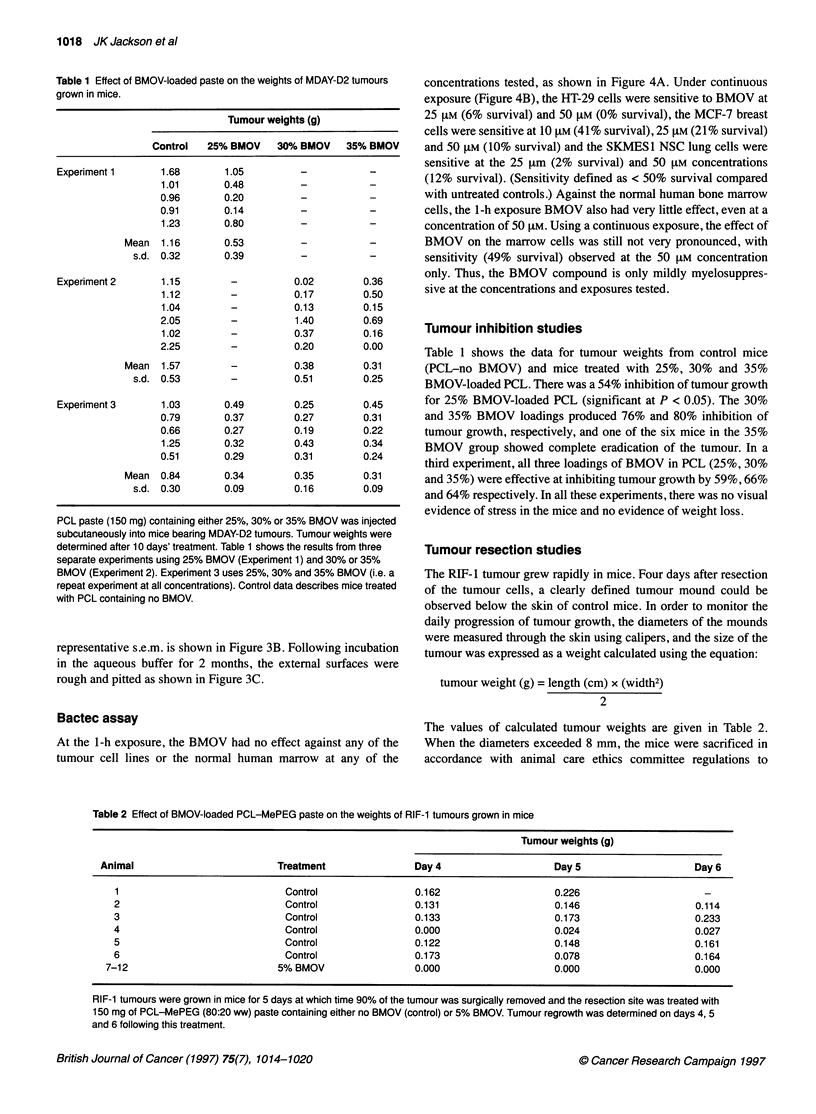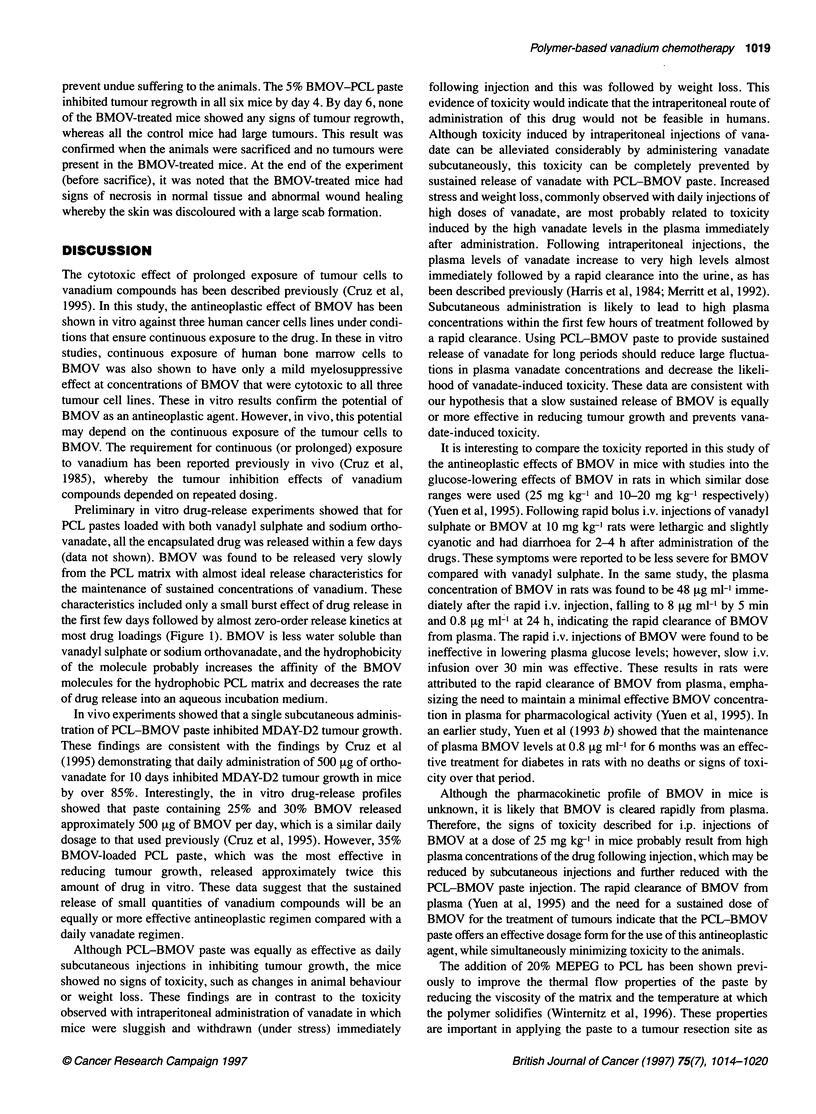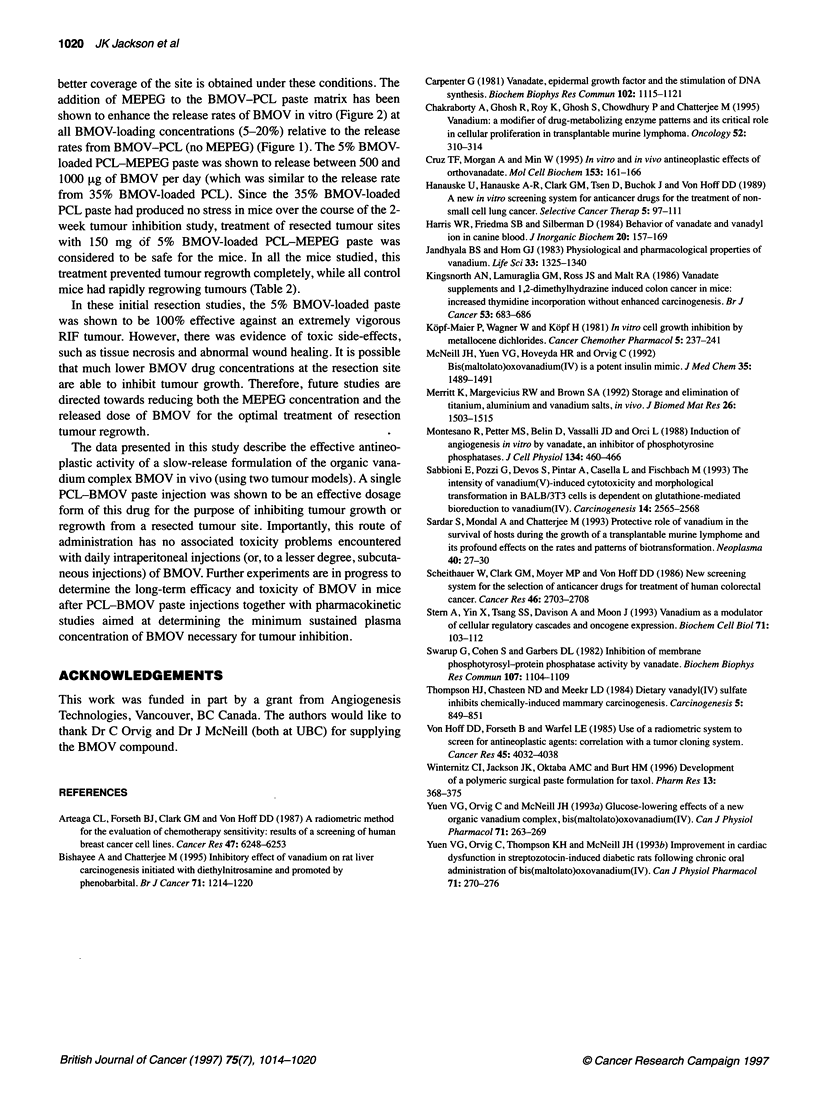# A polymer-based drug delivery system for the antineoplastic agent bis(maltolato)oxovanadium in mice.

**DOI:** 10.1038/bjc.1997.174

**Published:** 1997

**Authors:** J. K. Jackson, W. Min, T. F. Cruz, S. Cindric, L. Arsenault, D. D. Von Hoff, D. Degan, W. L. Hunter, H. M. Burt

**Affiliations:** Faculty of Pharmaceutical Sciences, University of British Columbia, Vancouver, Canada.

## Abstract

**Images:**


British Journal of Cancer (1997) 75(7), 1014–1020
© 1997 Cancer Research Campaign

### A polymer-based drug delivery system for the antineoplastic agent bis(maltolato)oxovanadium in mice

**JK Jackson[1], W Min[2], TF Cruz[2], S Cindric[3], L Arsenault[3], DD Von Hoff[4], D Degan[4], WL Hunter[5] and HM Burt[1]**

[1]Faculty of Pharmaceutical Sciences, 2146 East Mall, University of British Columbia, Vancouver, BC, Canada V6T 1Z3; [2] Connective Tissue Research Group, Samuel Lunenfeld Research Institute, Mount Sinai Hospital, 600 University Avenue, Toronto, Ontario, Canada M5G 1X5; [3] Pathology Department, Faculty of Health Sciences, McMaster University, Hamilton, Ontario, Canada, L8N 3Z5; [4] Institute for Drug Development, Cancer Therapy and Research Center, San Antonio, Texas, 78245-3217, USA; [5]Angiogenesis Technologies, 2120-1066 West Hastings Street, Vancouver, BC, Canada V6E 3X1

**Summary** Using vanadyl sulphate, sodium orthovanadate or bis(maltolato)oxovanadium (BMOV), Cruz TF, Morgan A, Min W (1995, *Mol Cell Biochem* **153:** 161–166) have recently demonstrated the antineoplastic effects of vanadium in mice. In this study, the antineoplastic effects of BMOV against human tumour cell lines was confirmed, and this effect was shown to depend on the prolonged exposure of the cells to the drug. We have investigated a polymeric drug delivery system for the sustained delivery of BMOV as an antineoplastic agent in mice. The objective was to design and evaluate an injectable polymer–BMOV paste that would act as a drug implant for the slow but sustained release of BMOV in the mice. In vitro studies showed that the biodegradable polymer poly (Ghlr epsilon ε-caprolactone) (PCL) released BMOV in a sustained manner with rates of drug release increasing with increased loading of the drug in the polymer. In vivo studies showed that PCL–BMOV paste implants produced a concentration-dependent inhibition of MDAY-D2 tumour growth via systemic drug delivery. Further in vivo studies showed that 5% BMOV-loaded PCL (containing 20% methoxypolyethylene glycol) was effective in preventing tumour regrowth of resected RIF tumour masses in mice when the PCL–BMOV paste was applied to the resected site for localized drug delivery. The results confirm the potential of vanadium as an antineoplastic agent and show that the injectable PCL–BMOV formulation releases a chemotheraputic dose of vanadium for the systemic treatment of whole tumours as well as the localized treatment of resected RIF tumours.

**Keywords:** polycaprolactone; vanadium; chemotherapy; resection

Vanadium is a potent in vitro inhibitor of various enzymes, such as $Na^+$, $K^+$-ATPase, $Ca^{2+}$ ATPase and various protein tyrosine phosphatases (Swarup et al, 1982; Jandhyala et al, 1983). Although normally present in plasma at submicromolar levels, increased concentrations of vanadium in plasma influence insulin sensitivity, vascular resistance and renal function (Jandhyala et al, 1983; McNeill et al, 1992). Mitogenic effects of vanadium have been reported previously (Carpenter, 1981 Kingsnorth et al, 1986; Montesano et al, 1988; Stern et al, 1993), and there is some evidence indicating a tumour-promoting potential of vanadium (Sabbioni et al, 1993; Stern et al, 1993; Chakraborty et al, 1995). In contrast to this, vanadium has been reported to have anticarcinogenic effects (Thompson et al, 1984; Bishayee et al, 1995) and to inhibit tumour cell growth (Kopf-Maier et al, 1981; Sardar et al, 1993).

The antineoplastic effects of vanadium have recently been confirmed both in vitro and in vivo (Cruz et al, 1995.). More than 85% inhibition of tumour growth was achieved following daily subcutaneous injections of 500 μg of orthovanadate into mice bearing the MDAY-D2 tumour grown in mice. Further studies showed that the vanadyl sulphate, orthovanadate and the organic vanadium complex, bis(maltolato)oxovanadium (BMOV) were all effective antineoplastic agents (personal communication, TF Cruz). It was noted that the mice showed some stress immediately after injection of the large doses of vanadium indicating that some of the toxicity is caused by the high initial plasma concentrations following vanadium administration. Continuous administration of vanadium compounds at lower plasma concentrations may prevent the toxicity associated with a bolus administration.

We have developed a slow-release polymeric surgical paste drug delivery system for the anti-cancer agent, taxol, for potential application at tumour resection sites to prevent local recurrence of disease (Winternitz, 1996). The poly (ε-caprolactone) (PCL)-based paste is applied to a tissue site in the molten state (about 55°C) where it solidifies rapidly to a hard waxy implant at 37°C.

In this study, we have investigated the suitability of the PCL paste as a delivery vehicle for the organic vanadate complex, BMOV. BMOV is a substantially more hydrophobic and less water-soluble form of vanadium (Yuen et al, 1993*a*) and is therefore preferable for the development of a controlled release drug delivery system.

The objectives of this work were to develop and characterize a PCL–BMOV formulation and determine its effectiveness both in the systemic treatment of MDAY-D2 tumour-bearing mice and in the local treatment of partially resected tumours in mice.

### MATERIALS AND METHODS

### Polymer formulations

Poly (ε-caprolactone) (molecular weight 20 000) (BPI Birmingham, AL, USA) and BMOV (a generous gift from Dr J McNeill) were weighed directly into a glass beaker in the appropriate proportions. In some formulations, methoxypolyethylene glycol (MEPEG)

*Received 19 August 1996*
*Revised 7 October 1996*
*Accepted 16 October 1996*

*Correspondence to:* HM Burt

(molecular weight 350) (Union Carbide, Danbury, CT, USA) was also added to the PCL and BMOV. The beaker and contents were warmed to 55°C with gentle stirring for 5 min until the BMOV was thoroughly dispersed in the molten polymer. The molten mix was then drawn into a prewarmed syringe and stored at 4°C until use.

### Drug-release studies

To tubes containing 15 ml of 10 mM phosphate-buffered saline (PBS, pH 7.4) and 100 μg $ml^{-1}$ bovine serum albumin (Fraction 5;Boehringer Mannheim, Germany) were added 150 mg disc-shaped slabs of PCL–BMOV paste. The tubes were sealed and tumbled end over end at 30 r.p.m. at 37°C. At appropriate times, the PCL–BMOV slab was allowed to settle under gravity for 5 min and all the supernatant was removed. The BMOV concentration was determined in the supernatants by measuring the absorbance at 256 nm (A256) and 276 nm (A276). The supernatant was replaced with 15 ml of fresh PBS and the tubes were retumbled. A linear calibration curve of BMOV concentration vs A256 or A276 was obtained using BMOV standards in the 0–25 μg $ml^{-1}$ range. The absorbance values at 256 nm or 276 nm of these standards were shown to be unaffected by storage in sealed tubes at 37°C for 2–3 days (the same conditions used for drug-release studies). At the end of the drug-release experiments, samples of the PCL–BMOV matrix were assayed for residual drug content by dissolution of a known dried weight of the matrix in 0.5 ml of dichloromethane (DCM) (Fisher). To this solution was added 50 ml of warm water (50°C) with mixing to evaporate the DCM, leaving the BMOV in water for spectrophotometric analysis (A256).

### Scanning electron microscopy (SEM)

Samples of the PCL–BMOV matrix that had been used in the 2-month drug-release experiments were examined using SEM. These samples were compared with freshly prepared control samples. Polymer samples were coated (60:40 gold:palladium) (Hummer Instruments, Technics, USA) and examined using a Hitachi (model F-2300) scanning electron microscope with an IBM data collection system.

### Human tumour cell lines

The HT-29 colon, MCF-7 breast and SKMES1 non-small-cell lung human tumour cell lines were obtained from the American Type Culture Collection. The HT-29 colon cell line was cultured in RPMI-1640 medium with 10% heat-inactivated fetal bovine serum (HIFBS); the MCF-7 breast cell line in Iscove's modified Eagle medium with 5% HIFBS plus $10^{-9}$ M insulin; and the SKMES1 lung cell line in Eagle minimal essential medium with 10% non-heat-inactivated FBS. The use of these cell lines to determine the anti-neoplastic activity of various agents has been described previously (Scheithauer et al, 1986; Arteaga et al, 1987; Hanauske, 1989).

### Normal human marrow cells

Normal human bone marrow (histologically negative for tumour cells) was obtained from patients who were to have bone marrow transplants for their solid tumours but died before the marrow was used. After centrifugation, the buffy coat was removed and cells were treated with lysis buffer and washed twice with and then resuspended in RPMI-1640 medium with 20% HIFBS. The cells were drawn through a 25G needle and counted.

### Radiometric (Bactec) system

The Bactec system (Johnston Laboratories, Towson, MD, USA) is based on a clinical instrument, which was developed to detect bacteria in blood cultures. The instrument has been used to screen for new antineoplastic agents (Von Hoff et al, 1985). This radiometric system is a rapid, semi-automated system that uses the inhibition of the conversion of [$^{14}$C]glucose to $^{14}CO_2$ as an index of cytotoxicity. The Bactec instrument automatically flushes out the $^{14}CO_2$ into an ion chamber where the signal of the radiolabelled $CO_2$ is changed into a proportional electrical signal or growth index value on a scale of 1 to 1000. For the continuous exposure, the tumour cells or normal marrow cells were added to 2 ml of the appropriate growth medium containing 2 μCi of [$^{14}$C]glucose plus BMOV at final concentrations of 0.01, 0.1,1, 10,25 and 50 uM and injected into 20-ml rubber-stoppered serum vials, which contained a mixture of 5% carbon dioxide and air, and incubated at 37°C for 24 days. For 1-h exposure, cells and BMOV at the same final concentrations used in the continuous exposure vials were incubated in 15-ml polypropylene conicals in a 37°C water bath for 1 h. The cells were then centrifuged and washed in medium, then resuspended in 2 ml of the appropriate growth medium containing 2 μCi of [$^{14}$C]glucose and injected into 20-ml rubber-stoppered serum vials, which contained a mixture of 5% carbon dioxide and air, and incubated at 37°C for 24 days. At days 6,9 and 12 for tumour cell lines and days 6,15 and 24 for marrow cells, the vials were removed and inserted into the Bactec instrument for determination of the amount of $^{14}CO_2$ produced by the cells upon metabolizing the [$^{14}$C]glucose. The growth index values of BMOV-treated cells were compared with the growth index values of non-treated cells and the percentage survival compared with untreated controls was calculated.

### Resected tumour studies

These experiments were carried out with animal care ethics committee approval at McMaster University, Hamilton, Ontario, Canada. Seven-week-old, male C3H/HeJ mice were used in these studies. RIF-1 (murine radiation-induced fibrosarcoma) cells were cultured in alpha-minimum essential medium (MEM) containing 10% FBS (Gibco, Canada). Cells were suspended in 1% Hanks' buffered salt solution (HBSS, pH 7.4) (Gibco, Canada) at a concentration of $1 \times 10^7$ cells per ml. Approximately 100 μl of these cells ($1 \times 10^6$ cells) was injected into the right flank of each mouse. The tumours were allowed to grow for 5 days (at which time the tumours ranged from 6 to 8 mm in diameter). At day 5, the mice were anaesthetized with a Ketamine–Rompom (70 mg $kg^{-1}$:10 mg $kg^{-1}$) combination (0.02 ml $g^{-1}$). An incision was made 5 mm from the tumour edge and approximately 90% of each tumour was removed and 150 mg of molten (50°C) PCL–MEPEG–BMOV or PCL–MEPEG alone (control) was extruded from a 500 μl syringe onto the entire surface of the resected tumour site. The PCL solidified within 30 s and the area was closed with 5–0 prolene sutures. The mice were examined on days 4, 5, 6 and 7. On each day, tumours were measured (long and short diameters) and images taken. When the tumours reached a maximum diameter of 9 mm, the mice were sacrificed and the tumour area was excised for future histological studies.

© Cancer Research Campaign 1997

**Figure 1** Time course of BMOV release from PCL (150-mg slabs). **(A)** Drug released (µg) or **(B)** percentage of drug remaining in slab. Initial loading of BMOV in PCL given by (○), 5%; (●), 10%; (△), 15%; (▲), 20%; (□), 25%; (■), 30% and (▽), 35%

**Figure 2** Time course of BMOV release from 150-mg slabs of PCL–MEPEG (80:20, ww) expressed as **(A)** drug released (µg) or **(B)** percentage drug remaining in slab. Initial loading of BMOV in PCL–MEPEG given by (○), 5%; (●), 10%; (△), 15% and (▲), 20%

### Tumour inhibition studies

These experiments were performed using 10-week-old DBA/2j female mice with animal ethics committee approval at the University of Toronto.

The MDAY-D2 haematopoetic cell line was obtained from Dr J Dennis, Mount Sinai Hospital, Toronto, Canada. These cells were plated or grown in suspension in Dulbecco's modified Eagle medium (DMEM) containing 5% FBS (Gibco, Canada). Each mouse was injected subcutaneously on the posterior lateral side with $4 \times 10^5$ cells in 100 µl of PBS. After 5 days' tumour growth, 150 mg of the PCL or PCL–BMOV molten paste was implanted in an area adjacent to the tumour site of each mouse. After 15 days, the mice were sacrificed, weighed and the tumours dissected and weighed.

Statistical significance was determined using the Student's T-test at $P < 0.05$

### RESULTS

### In vitro drug-release experiments

The release of BMOV from the PCL matrix is shown in Figure 1. Increasing the loading of BMOV from 5% to 35% in the PCL matrix increased the rate of drug release over the 2-month period (Figure 1A). At 35% BMOV loading, the release rate increased markedly. The release profiles for the 20% to 30% BMOV loadings showed an initial more rapid phase of drug release in the first 2 days followed by a controlled, almost zero-order, release over the next 2 months.

The drug-release profiles are also expressed in terms of the percentage of drug remaining in the pellet (Figure 1B). The percentage of BMOV remaining in the PCL matrix was almost identical at all time points for all the BMOV loadings up to (and including) 30%, so that between 65% and 80% of the original BMOV was still present in the matrix after 2 months. (Only the data for 25%, 30% and 35% BMOV loadings are shown in Figure 1B for clarity.) The percentage of drug remaining in the matrix decreased more rapidly at a loading of 35% BMOV, so that approximately 50% of the original BMOV was present in the matrix after 1 month. In order to verify the cumulative drug-release data, samples of the PCL–BMOV matrix were assayed for remaining drug content at the end of each drug-release experiment. The percentage of drug remaining at 70 days as determined by this residual assay was as follows: 67% ± 10% (5% BMOV), 56% ± 10% (10% BMOV), 80% ± 20% (15% BMOV), 84% ± 20% (20% BMOV), 85% ± 12% (25% BMOV), 77% ± 14% (30% BMOV) and 57% ± 15% (35% BMOV).

Figure 2 shows the effect of adding 20% MEPEG to the PCL matrix on the drug-release profiles for various loading concentrations of BMOV. The addition of MEPEG to the matrix increases the release rate of BMOV dramatically compared with the release

*© Cancer Research Campaign 1997*

**Figure 3** Scanning electron micrographs of (**A**) BMOV crystals, (**B**) surface morphology of the PCL slab containing 20% BMOV at the start of the drug-release experiment and (**C**) surface morphology of the PCL slab containing 20% BMOV at the end of the drug-release experiment (72 days in PBS)

**Figure 4** Effect of increasing concentration of BMOV on cell survival using (**A**) 1-h exposure of cells to BMOV or (**B**) continuous exposure to BMOV. Cells described by (○), HT-29 colon cells; (●), MCF-7 breast cells; (△), Skmes1 non-small-cell lung cancer cells and (▲), normal bone marrow cells of BMOV from the PCL alone (Figure 1.). More than 50% of the BMOV was released from the polymer matrix within 7 days at all BMOV loading concentrations. Residual analysis of the PCL–BMOV–MEPEG pellets gave the following values for the percentage of BMOV remaining at 7 days in the pellets: 24% ± 9% (5% BMOV), 25% ± 8% (10% BMOV), 22% ± 4% (15% BMOV), 27% ± 7% (20% BMOV).

### Scanning electron micrographs of PCL–BMOV matrices

Figure 3A shows the morphology of the BMOV crystals under high magnification. Figure 3B and C shows the morphology of the surface of the polymer–drug matrices both before and after the 2-month drug-release study in aqueous buffer. The PCL–BMOV matrices for 15%, 20% and 30% BMOV loadings were typically smooth on their external surfaces before the release study and a

**Table 1** Effect of BMOV-loaded paste on the weights of MDAY-D2 tumours grown in mice.

| | | Tumour weights (g) | | | |
|---|---|---|---|---|---|
| | | Control | 25% BMOV | 30% BMOV | 35% BMOV |
| Experiment 1 | | 1.68 | 1.05 | – | – |
| | | 1.01 | 0.48 | – | – |
| | | 0.96 | 0.20 | – | – |
| | | 0.91 | 0.14 | – | – |
| | | 1.23 | 0.80 | – | – |
| | Mean | 1.16 | 0.53 | – | – |
| | s.d. | 0.32 | 0.39 | – | – |
| Experiment 2 | | 1.15 | – | 0.02 | 0.36 |
| | | 1.12 | – | 0.17 | 0.50 |
| | | 1.04 | – | 0.13 | 0.15 |
| | | 2.05 | – | 1.40 | 0.69 |
| | | 1.02 | – | 0.37 | 0.16 |
| | | 2.25 | – | 0.20 | 0.00 |
| | Mean | 1.57 | – | 0.38 | 0.31 |
| | s.d. | 0.53 | – | 0.51 | 0.25 |
| Experiment 3 | | 1.03 | 0.49 | 0.25 | 0.45 |
| | | 0.79 | 0.37 | 0.27 | 0.31 |
| | | 0.66 | 0.27 | 0.19 | 0.22 |
| | | 1.25 | 0.32 | 0.43 | 0.34 |
| | | 0.51 | 0.29 | 0.31 | 0.24 |
| | Mean | 0.84 | 0.34 | 0.35 | 0.31 |
| | s.d. | 0.30 | 0.09 | 0.16 | 0.09 |

PCL paste (150 mg) containing either 25%, 30% or 35% BMOV was injected subcutaneously into mice bearing MDAY-D2 tumours. Tumour weights were determined after 10 days' treatment. Table 1 shows the results from three separate experiments using 25% BMOV (Experiment 1) and 30% or 35% BMOV (Experiment 2). Experiment 3 uses 25%, 30% and 35% BMOV (i.e. a repeat experiment at all concentrations). Control data describes mice treated with PCL containing no BMOV.

representative s.e.m. is shown in Figure 3B. Following incubation in the aqueous buffer for 2 months, the external surfaces were rough and pitted as shown in Figure 3C.

### Bactec assay

At the 1-h exposure, the BMOV had no effect against any of the tumour cell lines or the normal human marrow at any of the concentrations tested, as shown in Figure 4A. Under continuous exposure (Figure 4B), the HT-29 cells were sensitive to BMOV at 25 μM (6% survival) and 50 μM (0% survival), the MCF-7 breast cells were sensitive at 10 μM (41% survival), 25 μM (21% survival) and 50 μM (10% survival) and the SKMES1 NSC lung cells were sensitive at the 25 μm (2% survival) and 50 μM concentrations (12% survival). (Sensitivity defined as < 50% survival compared with untreated controls.) Against the normal human bone marrow cells, the 1-h exposure BMOV also had very little effect, even at a concentration of 50 μM. Using a continuous exposure, the effect of BMOV on the marrow cells was still not very pronounced, with sensitivity (49% survival) observed at the 50 μM concentration only. Thus, the BMOV compound is only mildly myelosuppressive at the concentrations and exposures tested.

### Tumour inhibition studies

Table 1 shows the data for tumour weights from control mice (PCL–no BMOV) and mice treated with 25%, 30% and 35% BMOV-loaded PCL. There was a 54% inhibition of tumour growth for 25% BMOV-loaded PCL (significant at $P < 0.05$). The 30% and 35% BMOV loadings produced 76% and 80% inhibition of tumour growth, respectively, and one of the six mice in the 35% BMOV group showed complete eradication of the tumour. In a third experiment, all three loadings of BMOV in PCL (25%, 30% and 35%) were effective at inhibiting tumour growth by 59%, 66% and 64% respectively. In all these experiments, there was no visual evidence of stress in the mice and no evidence of weight loss.

### Tumour resection studies

The RIF-1 tumour grew rapidly in mice. Four days after resection of the tumour cells, a clearly defined tumour mound could be observed below the skin of control mice. In order to monitor the daily progression of tumour growth, the diameters of the mounds were measured through the skin using calipers, and the size of the tumour was expressed as a weight calculated using the equation:

$$\text{tumour weight (g)} = \frac{\text{length (cm)} \times (\text{width}^2)}{2}$$

The values of calculated tumour weights are given in Table 2. When the diameters exceeded 8 mm, the mice were sacrificed in accordance with animal care ethics committee regulations to

**Table 2** Effect of BMOV-loaded PCL–MePEG paste on the weights of RIF-1 tumours grown in mice

| | | Tumour weights (g) | | |
|---|---|---|---|---|
| Animal | Treatment | Day 4 | Day 5 | Day 6 |
| 1 | Control | 0.162 | 0.226 | – |
| 2 | Control | 0.131 | 0.146 | 0.114 |
| 3 | Control | 0.133 | 0.173 | 0.233 |
| 4 | Control | 0.000 | 0.024 | 0.027 |
| 5 | Control | 0.122 | 0.148 | 0.161 |
| 6 | Control | 0.173 | 0.078 | 0.164 |
| 7–12 | 5% BMOV | 0.000 | 0.000 | 0.000 |

RIF-1 tumours were grown in mice for 5 days at which time 90% of the tumour was surgically removed and the resection site was treated with 150 mg of PCL–MePEG (80:20 ww) paste containing either no BMOV (control) or 5% BMOV. Tumour regrowth was determined on days 4, 5 and 6 following this treatment.

© Cancer Research Campaign 1997

prevent undue suffering to the animals. The 5% BMOV–PCL paste inhibited tumour regrowth in all six mice by day 4. By day 6, none of the BMOV-treated mice showed any signs of tumour regrowth, whereas all the control mice had large tumours. This result was confirmed when the animals were sacrificed and no tumours were present in the BMOV-treated mice. At the end of the experiment (before sacrifice), it was noted that the BMOV-treated mice had signs of necrosis in normal tissue and abnormal wound healing whereby the skin was discoloured with a large scab formation.

### DISCUSSION

The cytotoxic effect of prolonged exposure of tumour cells to vanadium compounds has been described previously (Cruz et al, 1995). In this study, the antineoplastic effect of BMOV has been shown in vitro against three human cancer cells lines under conditions that ensure continuous exposure to the drug. In these in vitro studies, continuous exposure of human bone marrow cells to BMOV was also shown to have only a mild myelosuppressive effect at concentrations of BMOV that were cytotoxic to all three tumour cell lines. These in vitro results confirm the potential of BMOV as an antineoplastic agent. However, in vivo, this potential may depend on the continuous exposure of the tumour cells to BMOV. The requirement for continuous (or prolonged) exposure to vanadium has been reported previously in vivo (Cruz et al, 1985), whereby the tumour inhibition effects of vanadium compounds depended on repeated dosing.

Preliminary in vitro drug-release experiments showed that for PCL pastes loaded with both vanadyl sulphate and sodium orthovanadate, all the encapsulated drug was released within a few days (data not shown). BMOV was found to be released very slowly from the PCL matrix with almost ideal release characteristics for the maintenance of sustained concentrations of vanadium. These characteristics included only a small burst effect of drug release in the first few days followed by almost zero-order release kinetics at most drug loadings (Figure 1). BMOV is less water soluble than vanadyl sulphate or sodium orthovanadate, and the hydrophobicity of the molecule probably increases the affinity of the BMOV molecules for the hydrophobic PCL matrix and decreases the rate of drug release into an aqueous incubation medium.

In vivo experiments showed that a single subcutaneous administration of PCL–BMOV paste inhibited MDAY-D2 tumour growth. These findings are consistent with the findings by Cruz et al (1995) demonstrating that daily administration of 500 μg of orthovanadate for 10 days inhibited MDAY-D2 tumour growth in mice by over 85%. Interestingly, the in vitro drug-release profiles showed that paste containing 25% and 30% BMOV released approximately 500 μg of BMOV per day, which is a similar daily dosage to that used previously (Cruz et al, 1995). However, 35% BMOV-loaded PCL paste, which was the most effective in reducing tumour growth, released approximately twice this amount of drug in vitro. These data suggest that the sustained release of small quantities of vanadium compounds will be an equally or more effective antineoplastic regimen compared with a daily vanadate regimen.

Although PCL–BMOV paste was equally as effective as daily subcutaneous injections in inhibiting tumour growth, the mice showed no signs of toxicity, such as changes in animal behaviour or weight loss. These findings are in contrast to the toxicity observed with intraperitoneal administration of vanadate in which mice were sluggish and withdrawn (under stress) immediately following injection and this was followed by weight loss. This evidence of toxicity would indicate that the intraperitoneal route of administration of this drug would not be feasible in humans. Although toxicity induced by intraperitoneal injections of vanadate can be alleviated considerably by administering vanadate subcutaneously, this toxicity can be completely prevented by sustained release of vanadate with PCL–BMOV paste. Increased stress and weight loss, commonly observed with daily injections of high doses of vanadate, are most probably related to toxicity induced by the high vanadate levels in the plasma immediately after administration. Following intraperitoneal injections, the plasma levels of vanadate increase to very high levels almost immediately followed by a rapid clearance into the urine, as has been described previously (Harris et al, 1984; Merritt et al, 1992). Subcutaneous administration is likely to lead to high plasma concentrations within the first few hours of treatment followed by a rapid clearance. Using PCL–BMOV paste to provide sustained release of vanadate for long periods should reduce large fluctuations in plasma vanadate concentrations and decrease the likelihood of vanadate-induced toxicity. These data are consistent with our hypothesis that a slow sustained release of BMOV is equally or more effective in reducing tumour growth and prevents vanadate-induced toxicity.

It is interesting to compare the toxicity reported in this study of the antineoplastic effects of BMOV in mice with studies into the glucose-lowering effects of BMOV in rats in which similar dose ranges were used (25 mg $kg^{-1}$ and 10–20 mg $kg^{-1}$ respectively) (Yuen et al, 1995). Following rapid bolus i.v. injections of vanadyl sulphate or BMOV at 10 mg $kg^{-1}$ rats were lethargic and slightly cyanotic and had diarrhoea for 2–4 h after administration of the drugs. These symptoms were reported to be less severe for BMOV compared with vanadyl sulphate. In the same study, the plasma concentration of BMOV in rats was found to be 48 μg $ml^{-1}$ immediately after the rapid i.v. injection, falling to 8 μg $ml^{-1}$ by 5 min and 0.8 μg $ml^{-1}$ at 24 h, indicating the rapid clearance of BMOV from plasma. The rapid i.v. injections of BMOV were found to be ineffective in lowering plasma glucose levels; however, slow i.v. infusion over 30 min was effective. These results in rats were attributed to the rapid clearance of BMOV from plasma, emphasizing the need to maintain a minimal effective BMOV concentration in plasma for pharmacological activity (Yuen et al, 1995). In an earlier study, Yuen et al (1993 *b*) showed that the maintenance of plasma BMOV levels at 0.8 μg $ml^{-1}$ for 6 months was an effective treatment for diabetes in rats with no deaths or signs of toxicity over that period.

Although the pharmacokinetic profile of BMOV in mice is unknown, it is likely that BMOV is cleared rapidly from plasma. Therefore, the signs of toxicity described for i.p. injections of BMOV at a dose of 25 mg $kg^{-1}$ in mice probably result from high plasma concentrations of the drug following injection, which may be reduced by subcutaneous injections and further reduced with the PCL–BMOV paste injection. The rapid clearance of BMOV from plasma (Yuen at al, 1995) and the need for a sustained dose of BMOV for the treatment of tumours indicate that the PCL–BMOV paste offers an effective dosage form for the use of this antineoplastic agent, while simultaneously minimizing toxicity to the animals.

The addition of 20% MEPEG to PCL has been shown previously to improve the thermal flow properties of the paste by reducing the viscosity of the matrix and the temperature at which the polymer solidifies (Winternitz et al, 1996). These properties are important in applying the paste to a tumour resection site as

© Cancer Research Campaign 1997

better coverage of the site is obtained under these conditions. The addition of MEPEG to the BMOV–PCL paste matrix has been shown to enhance the release rates of BMOV in vitro (Figure 2) at all BMOV-loading concentrations (5–20%) relative to the release rates from BMOV–PCL (no MEPEG) (Figure 1). The 5% BMOV-loaded PCL–MEPEG paste was shown to release between 500 and 1000 µg of BMOV per day (which was similar to the release rate from 35% BMOV-loaded PCL). Since the 35% BMOV-loaded PCL paste had produced no stress in mice over the course of the 2-week tumour inhibition study, treatment of resected tumour sites with 150 mg of 5% BMOV-loaded PCL–MEPEG paste was considered to be safe for the mice. In all the mice studied, this treatment prevented tumour regrowth completely, while all control mice had rapidly regrowing tumours (Table 2).

In these initial resection studies, the 5% BMOV-loaded paste was shown to be 100% effective against an extremely vigorous RIF tumour. However, there was evidence of toxic side-effects, such as tissue necrosis and abnormal wound healing. It is possible that much lower BMOV drug concentrations at the resection site are able to inhibit tumour growth. Therefore, future studies are directed towards reducing both the MEPEG concentration and the released dose of BMOV for the optimal treatment of resection tumour regrowth.

The data presented in this study describe the effective antineoplastic activity of a slow-release formulation of the organic vanadium complex BMOV in vivo (using two tumour models). A single PCL–BMOV paste injection was shown to be an effective dosage form of this drug for the purpose of inhibiting tumour growth or regrowth from a resected tumour site. Importantly, this route of administration has no associated toxicity problems encountered with daily intraperitoneal injections (or, to a lesser degree, subcutaneous injections) of BMOV. Further experiments are in progress to determine the long-term efficacy and toxicity of BMOV in mice after PCL–BMOV paste injections together with pharmacokinetic studies aimed at determining the minimum sustained plasma concentration of BMOV necessary for tumour inhibition.

### ACKNOWLEDGEMENTS

This work was funded in part by a grant from Angiogenesis Technologies, Vancouver, BC Canada. The authors would like to thank Dr C Orvig and Dr J McNeill (both at UBC) for supplying the BMOV compound.

### REFERENCES

Arteaga CL, Forseth BJ, Clark GM and Von Hoff DD (1987) A radiometric method for the evaluation of chemotherapy sensitivity: results of a screening of human breast cancer cell lines. *Cancer Res* **47:** 6248–6253

Bishayee A and Chatterjee M (1995) Inhibitory effect of vanadium on rat liver carcinogenesis initiated with diethylnitrosamine and promoted by phenobarbital. *Br J Cancer* **71:** 1214–1220

Carpenter G (1981) Vanadate, epidermal growth factor and the stimulation of DNA synthesis. *Biochem Biophys Res Commun* **102:** 1115–1121

Chakraborty A, Ghosh R, Roy K, Ghosh S, Chowdhury P and Chatterjee M (1995) Vanadium: a modifier of drug-metabolizing enzyme patterns and its critical role in cellular proliferation in transplantable murine lymphoma. *Oncology* **52:** 310–314

Cruz TF, Morgan A and Min W (1995) *In vitro* and *in vivo* antineoplastic effects of orthovanadate. *Mol Cell Biochem* **153:** 161–166

Hanauske U, Hanauske A-R, Clark GM, Tsen D, Buchok J and Von Hoff DD (1989) A new *in vitro* screening system for anticancer drugs for the treatment of non-small cell lung cancer. *Selective Cancer Therap* **5:** 97–111

Harris WR, Friedma SB and Silberman D (1984) Behavior of vanadate and vanadyl ion in canine blood. *J Inorganic Biochem* **20:** 157–169

Jandhyala BS and Hom GJ (1983) Physiological and pharmacological properties of vanadium. *Life Sci* **33:** 1325–1340

Kingsnorth AN, Lamuraglia GM, Ross JS and Malt RA (1986) Vanadate supplements and 1,2-dimethylhydrazine induced colon cancer in mice: increased thymidine incorporation without enhanced carcinogenesis. *Br J Cancer* **53:** 683–686

Köpf-Maier P, Wagner W and Köpf H (1981) *In vitro* cell growth inhibition by metallocene dichlorides. *Cancer Chemother Pharmacol* **5:** 237–241

McNeill JH, Yuen VG, Hoveyda HR and Orvig C (1992) Bis(maltolato)oxovanadium(IV) is a potent insulin mimic. *J Med Chem* **35:** 1489–1491

Merritt K, Margevicius RW and Brown SA (1992) Storage and elimination of titanium, aluminium and vanadium salts, *in vivo*. *J Biomed Mat Res* **26:** 1503–1515

Montesano R, Petter MS, Belin D, Vassalli JD and Orci L (1988) Induction of angiogenesis *in vitro* by vanadate, an inhibitor of phosphotyrosine phosphatases. *J Cell Physiol* **134:** 460–466

Sabbioni E, Pozzi G, Devos S, Pintar A, Casella L and Fischbach M (1993) The intensity of vanadium(V)-induced cytotoxicity and morphological transformation in BALB/3T3 cells is dependent on glutathione-mediated bioreduction to vanadium(IV). *Carcinogenesis* **14:** 2565–2568

Sardar S, Mondal A and Chatterjee M (1993) Protective role of vanadium in the survival of hosts during the growth of a transplantable murine lymphome and its profound effects on the rates and patterns of biotransformation. *Neoplasma* **40:** 27–30

Scheithauer W, Clark GM, Moyer MP and Von Hoff DD (1986) New screening system for the selection of anticancer drugs for treatment of human colorectal cancer. *Cancer Res* **46:** 2703–2708

Stern A, Yin X, Tsang SS, Davison A and Moon J (1993) Vanadium as a modulator of cellular regulatory cascades and oncogene expression. *Biochem Cell Biol* **71:** 103–112

Swarup G, Cohen S and Garbers DL (1982) Inhibition of membrane phosphotyrosyl–protein phosphatase activity by vanadate. *Biochem Biophys Res Commun* **107:** 1104–1109

Thompson HJ, Chasteen ND and Meekr LD (1984) Dietary vanadyl(IV) sulfate inhibits chemically-induced mammary carcinogenesis. *Carcinogenesis* **5:** 849–851

Von Hoff DD, Forseth B and Warfel LE (1985) Use of a radiometric system to screen for antineoplastic agents: correlation with a tumor cloning system. *Cancer Res* **45:** 4032–4038

Winternitz CI, Jackson JK, Oktaba AMC and Burt HM (1996) Development of a polymeric surgical paste formulation for taxol. *Pharm Res* **13:** 368–375

Yuen VG, Orvig C and McNeill JH (1993*a*) Glucose-lowering effects of a new organic vanadium complex, bis(maltolato)oxovanadium(IV). *Can J Physiol Pharmacol* **71:** 263–269

Yuen VG, Orvig C, Thompson KH and McNeill JH (1993*b*) Improvement in cardiac dysfunction in streptozotocin-induced diabetic rats following chronic oral administration of bis(maltolato)oxovanadium(IV). *Can J Physiol Pharmacol* **71:** 270–276

© Cancer Research Campaign 1997